# A novel three-dimensional magnetic particle imaging system based on the frequency mixing for the point-of-care diagnostics

**DOI:** 10.1038/s41598-020-68864-9

**Published:** 2020-07-16

**Authors:** Seung-Min Choi, Jae-Chan Jeong, Jinsun Kim, Eul-Gyoon Lim, Chang-beom Kim, Sang-Jin Park, Dae-Yong Song, Hans-Joachim Krause, Hyobong Hong, In So Kweon

**Affiliations:** 1 Artificial Intelligence Research Laboratory, Electronics and Telecommunications Research Institute (ETRI), Daejeon, Republic of Korea; 2 Division of Future Vehicle, Korea Advanced Institute of Science and Technology (KAIST), Daejeon, Republic of Korea; 3 Department of Anatomy and Neuroscience, School of Medicine, Eulji University, Daejeon, Republic of Korea; 4 Institute of Biological Information Processing, Bioelectronics (IBI-3), Forschungszentrum Jülich, Jülich, Germany

**Keywords:** Nanoscience and technology, Biomedical engineering, Electrical and electronic engineering

## Abstract

The magnetic particle imaging (MPI) is a technology that can image the concentrations of the superparamagnetic iron oxide nanoparticles (SPIONs) which can be used in biomedical diagnostics and therapeutics as non-radioactive tracers. We proposed a point-of-care testing MPI system (PoCT-MPI) that can be used for preclinical use for imaging small rodents (mice) injected with SPIONs not only in laboratories, but also at emergency sites far from laboratories. In particular, we applied a frequency mixing magnetic detection method to the PoCT-MPI, and proposed a hybrid field free line generator to reduce the power consumption, size and weight of the system. The PoCT-MPI is $20 \times 33 \times 45\,{\hbox{cm}}^3$ in size and weighs less than 100 kg. It can image a three-dimensional distribution of SPIONs injected into a biosample with less than 120 Wh of power consumption. Its detection limit is $0.13\,\upmu {\hbox{L}}$, 10 mg/mL, $1.3\,\upmu {\hbox{g}}$ (Fe).

## Introduction

MPI converts the nonlinear magnetization characteristics of SPIONs into signals to image concentration of SPIONs. SPIONs saturates when being exposed to magnetic fields of sufficient strength. If a periodic magnetic field waveform with sufficiently large intensity is applied to the SPIONs as the modulation field, harmonics with multiples of frequency are observed because their magnetization curves are nonlinear. Then, these harmonic waveforms can be used as a signal to distinguish SPIONs from other materials^1^. SPIONs, in addition, can be applied in biomedical diagnostics and therapeutics, because they can be conjugated with various biomolecules (*e.g.*, antibodies, lipids, proteins, *etc.*) which can specifically bind on the surface of the target diseased cells or organs. Therefore, SPIONs with biomolecules are used as tracers to promote the selective imaging of target symptom in MPI systems. Because of its potential as a biomedical diagnostic device, much research has been published on MPI in recent decades. Gleich *et al.*^1^ had shown for the first time that three dimensional spatial scanning is possible by building and shifting an field free point (FFP), a kind of selection field which saturates the magnetic field everywhere except for the region of interest. On the other hand, Krause *et al.*^2^ introduced FMMD method which applies an alternating current (AC) magnetic field of two frequency instead of using one to improve the signal to noise ratio (SNR) characteristics of the measured signal. Then, Hong *et al.*^3^ and Kim *et al.*^4^ imaged the two-dimensional (2D) concentration of SPIONs using 2D FMMD devices. In addition, lot of scientist have tried to enhance the sensitivity of 3D MPI systems^5–10^. In particular, Goodwill *et al.* proposed the Projection MPI introducing x-space^7^. Then they successfully implemented 3D MPI systems using two circular magnets and four Direct Current (DC) coil pairs^8^. In detail, these coils are used to electronically shift the FFP generated by NdFeB permanent magnets generating gradient fields of $7 \times 3.5 \times 3.5$ T/m. It can scans sophisticated samples of $45 \times 120\,{\hbox{mm}}^2$ size by spatial resolution of 2 mm consuming 30 KW of power for an operation. Zheng *et al.*^9,11^ imaged vitro tagged cells in a pellet using 20 seconds projection MPI scan and reconstructed image which showed their MPI can image 5.4 ng (Fe) particles in cells. Yu *et al.*^12,13^ achieved 3 ng (Fe)/voxel of detection limit and $1 \times 1 \times 0.7$ mm of spatial resolution using stronger gradients improving from 2.35 T/m to 6.3 T/m for preclinical imaging which are the best performance so far.

Next, in terms of the power consumption of MPI devices, the result of M.Graeser^10^ is known as the best, because they succeeded in imaging 134 mL, 14.7 ng/mL (Fe), $2\,\upmu \hbox{g}$ (Fe) of SPIONs with a hydrodynamic diameter of 130 nm with just 240 Wh of power dissipation.

In the research introduced so far, they can have lots of advantages in terms of sensitivity and spatial resoultion for the preclinical imaging applications. However, as the demands for remote medical diagnosis technologies are increasing due to social needs such as aging and medical support in underdeveloped areas^14–16^, it is necessary to reduce the power consumption, size and weight of the MPI system while preserving its sensitivity for the PoCT preclinical MPI application.

In this study, novel attempts were made to overcome the problems of size reduction, heat dissipation, and SNR improvement for a portable low power MPI system. First, the FMMD method was introduced into a sensor to measure the concentration of the SPIONs in the new MPI system, PoCT-MPI. Since the sensor based on FMMD method combines magnetic field transmission, signal reception and processing features into a single device, it significantly reduced the power consumption and the size of the PoCT-MPI. Second, in order to reduce the weight and size of the selection field generator, we proposed a hybrid type of FFL generator composed of NdFeB permanent magnets and a coil. It performs the spatial encoder function for PoCT-MPI. This hybrid structure allows the sample to be inserted into the PoCT-MPI while minimizing power consumption. Finally, Dynamic Power Management (DPM)^17^ is applied to allow MPI equipment to operate without an external active cooling system for decreasing coil’s temperature.

## Results

### System overview

**Figure 1:**
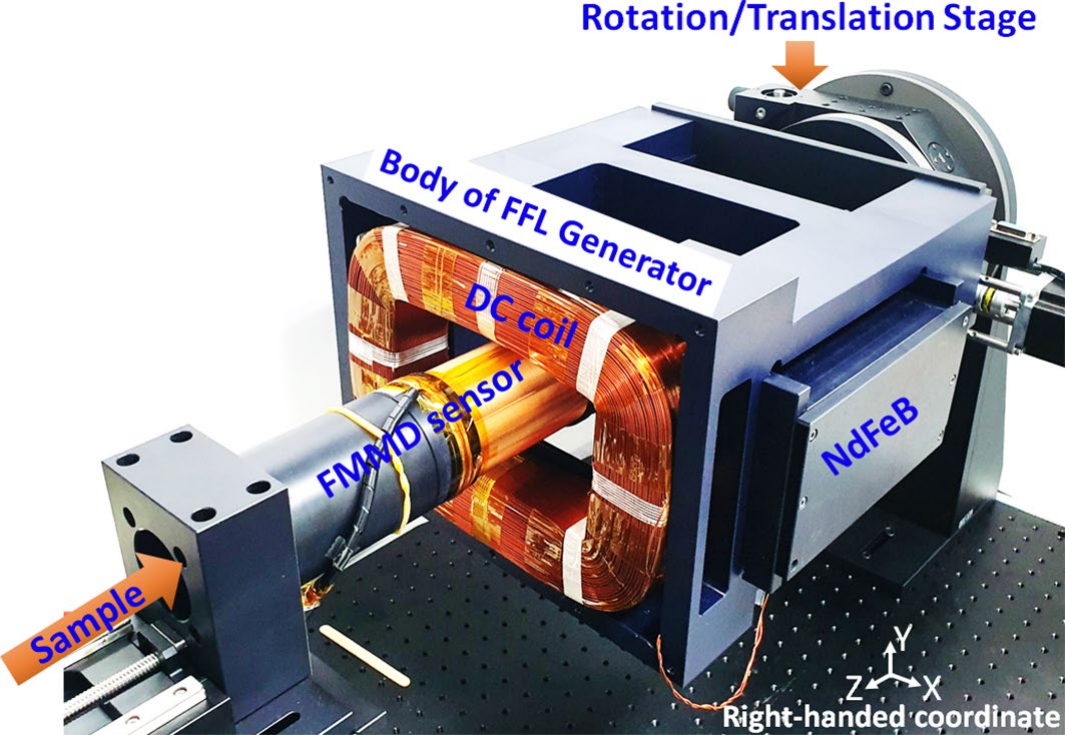
The proposed PoCT-MPI.

The PoCT-MPI consists of the FMMD sensor and the hybrid FFL generator as shown in Fig. 1. Note that the right-handed coordinate was used for all figures in this paper.

**Figure 2:**
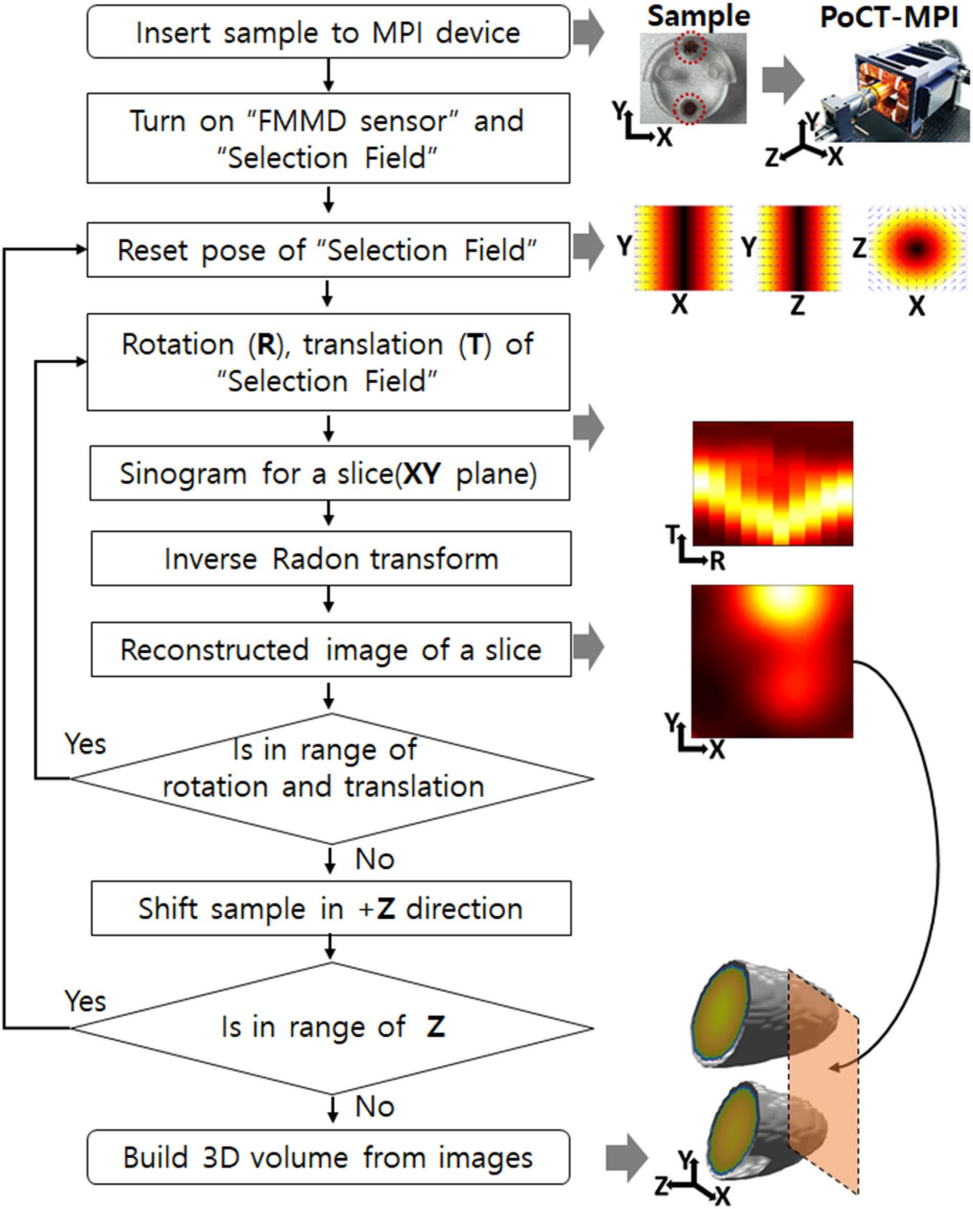
Operation of the PoCT-MPI.

The FMMD sensor detects the signal of all the SPIONs in the sample. Then, spatial encoding is possible by introducing an FFL selection field on it. In particular, we proposed a hybrid type FFL generator consisting of magnets and coil to decrease the size and power consumption of the PoCT-MPI. Then, the FFL generator was mounted on a moving stage to build sinograms of slices by performing Radon projections^18^. Next, the generated sinograms were reconstructed into the 2D images through inverse Radon transform^19^. Finally, the 2D images are merged into a 3D scalar volume image and then reconstructed into a 2D image in any direction desired by the user.

Figure 2 shows an operation process of the PoCT-MPI. After the sample is loaded into the PoCT-MPI, the FMMD sensor and FFL generator are activated. The sinogram is generated using the signal measured by the FMMD sensor during rotation and linear motion of the FFL generator. The sample moves in units of resolution in the Z direction, and a sinogram is generated at each location. Then, the measured sinograms are reconstructed into 2D images through the inverse Radon transform process. Finally, reconstructed 2D images are merged into 3D volume image.

### Simulation and experiment

**Figure 3:**
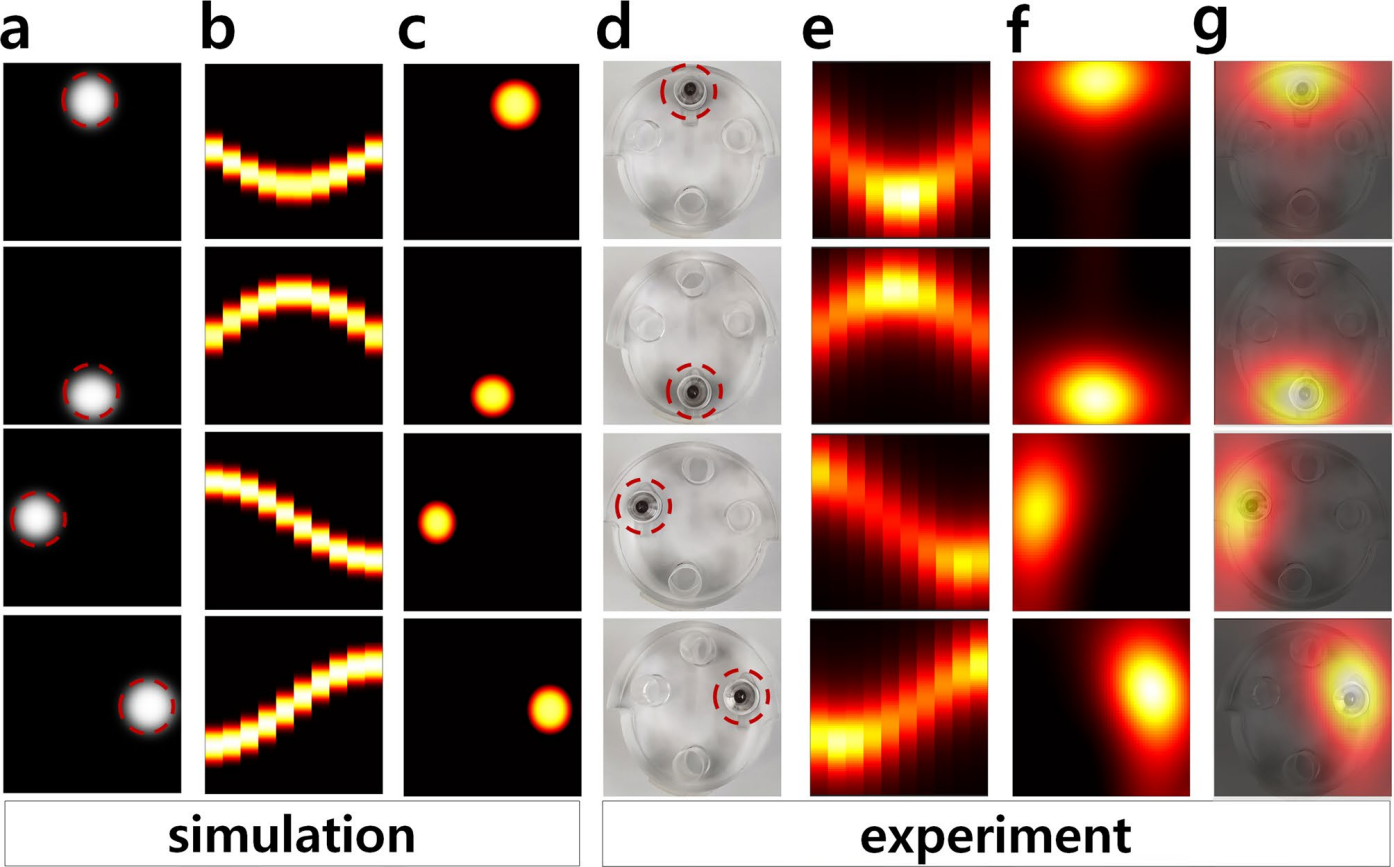
The result of scanning the samples(in dotted red circles) located in four positions of top, bottom, left and right using PoCT-MPI. simulation: (**a**) samples, (**b**) sinograms, (**c**) recontructed images, experiment(PoCT-MPI) : (**d**) samples, (**e**) sinograms, (**f**), recontructed images, (**g**) superimposed images (**d** + **f**).

We compared the experimental scan results of the PoCT-MPI with the simulation results using open source library^19^ as shown in Fig. 3. First, SPIONs were injected into the upper, lower, left, and right sides of the acrylic resin sample holder as shown in Fig. 3d similar to the samples of simulation (a). Note that (a) are virtual samples created with 0 and 1 for simulation, so they does not exactly match the actual samples (d) in Fig. 3. Therefore, it is necessary to focus on whether the sinograms are generated correctly and the images are reconstructed according to the positions of the samples. As a result of 2D scan using PoCT-MPI, we obtained sinograms (e) and reconstructed images(f) similar to them (b) and (c) of simulation as shown in Fig. 3. By superimposing the real samples (d) and the reconstructed images (f), we can see that the location matches with an error of mm level (hole of holder is 5 mm) as shown in Fig. 3g.

### Sensitivity and resolution

To measure the sensitivity, test tubes containing various amounts of SPIONs from 0.13 to 5 $\upmu \hbox{L}$ were loaded in the PoCT-MPI and were visualized to the reconstructed images. The SPIONs used in this experiment were synomag-d of Micromod in which concentrations of the solid and the iron (Fe) in suspension are 25 mg/mL and 10 mg/mL, respectively.

**Figure 4:**
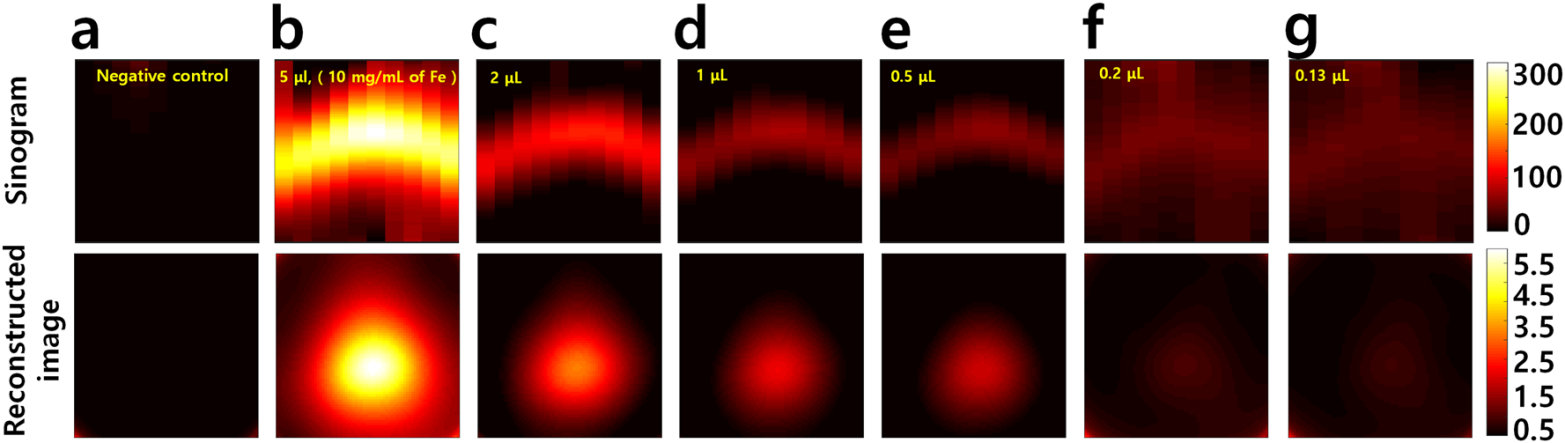
The sensitivity of the PoCT-MPI. The iron concentrations are 10 mg/mL. (**a**) negative control, (**b**) 5 $\upmu \hbox{L}$, 50 $\upmu \hbox{g}$ (Fe), (**c**) 2 $\upmu \hbox{L}$, 20 $\upmu \hbox{g}$ (Fe), (**d**) 1 $\upmu \hbox{L}$, 10 $\upmu \hbox{g}$ (Fe), (**e**) 0.5 $\upmu \hbox{L}$, 5 $\upmu \hbox{g}$ (Fe), (**f**) 0.2 $\upmu \hbox{L}$, 2 $\upmu \hbox{g}$ (Fe), (**g**) 0.13 $\upmu \hbox{L}$, 1.3 $\upmu \hbox{g}$ (Fe).

**Figure 5:**
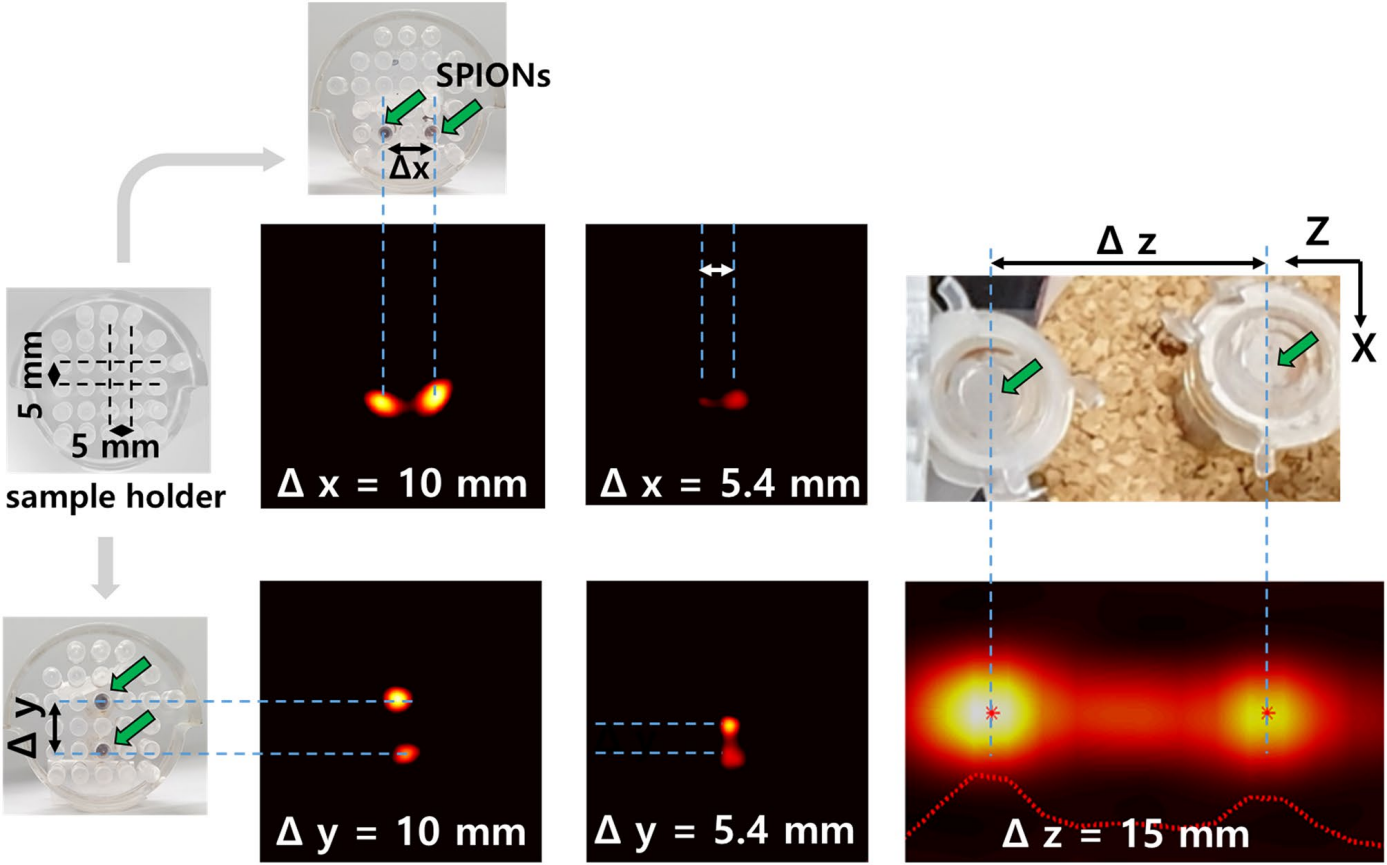
The spatial resolution of the PoCT-MPI.

As shown in Fig. 4, the minimal volume of suspension containing SPIONs was 0.13 $\upmu \hbox{L}$ in which the iron (Fe) content was 1.3 $\upmu \hbox{g}$. It was possible to determine the sample without artifacts starting from 5 $\upmu \hbox{L}$5 down to 0.5 $\upmu \hbox{L}$. In 0.2 $\upmu \hbox{L}$ and 0.13 $\upmu \hbox{L}$, some noise and artifacts appeared, but the presence of the sample can be distinguished compared to the negative control, Fig. 4a. Therefore, it can be said that the detection limit of the PoCT-MPI is about 0.13 $\upmu \hbox{L}$ (10 mg/mL of Fe), 1.3 $\upmu \hbox{g}$ (Fe).

Next, to measure the spatial resolution for X, Y direction, about 2 $\upmu \hbox{L}$ of SPIONs (for X, Y) were filled in capillary tubes. And for the Z direction, PCR tubes containing 10 $\upmu \hbox{L}$ of SPIONs were used as shown in Fig. 5. Note that we did not use 0.13 $\upmu \hbox{L}$, the lowest amount of sensitivity observed with some noise, but used an appropriate volume to verify performance in typical applications. Then, we made sample holders that can mount cpillary tubes in 5 mm increments for the measuring X, Y resolution as shown in left-top of Fig. 5. The Z direction was measured by fixing the sample on a wooden holder as shown in right-top of Fig. 5. Green arrows indicate SPIONs contained in the capillary tube. In Fig. 5, when injecting the suspension into the capillary tube using capillary pressure, it is difficult to disperse the SPIONs uniformly in the Z-axis direction, and as a result, the peak brightness or size is measured differently. However, since the peaks are observed separately, this experiment showed the conservative resolution of the equipment. As results, the spatial resolutions can be said 5.4 mm, 5.4 mm and 15 mm for $\Delta X$, $\Delta Y$ and $\Delta Z$, respectively as shown in Fig. 5.

**Figure 6:**
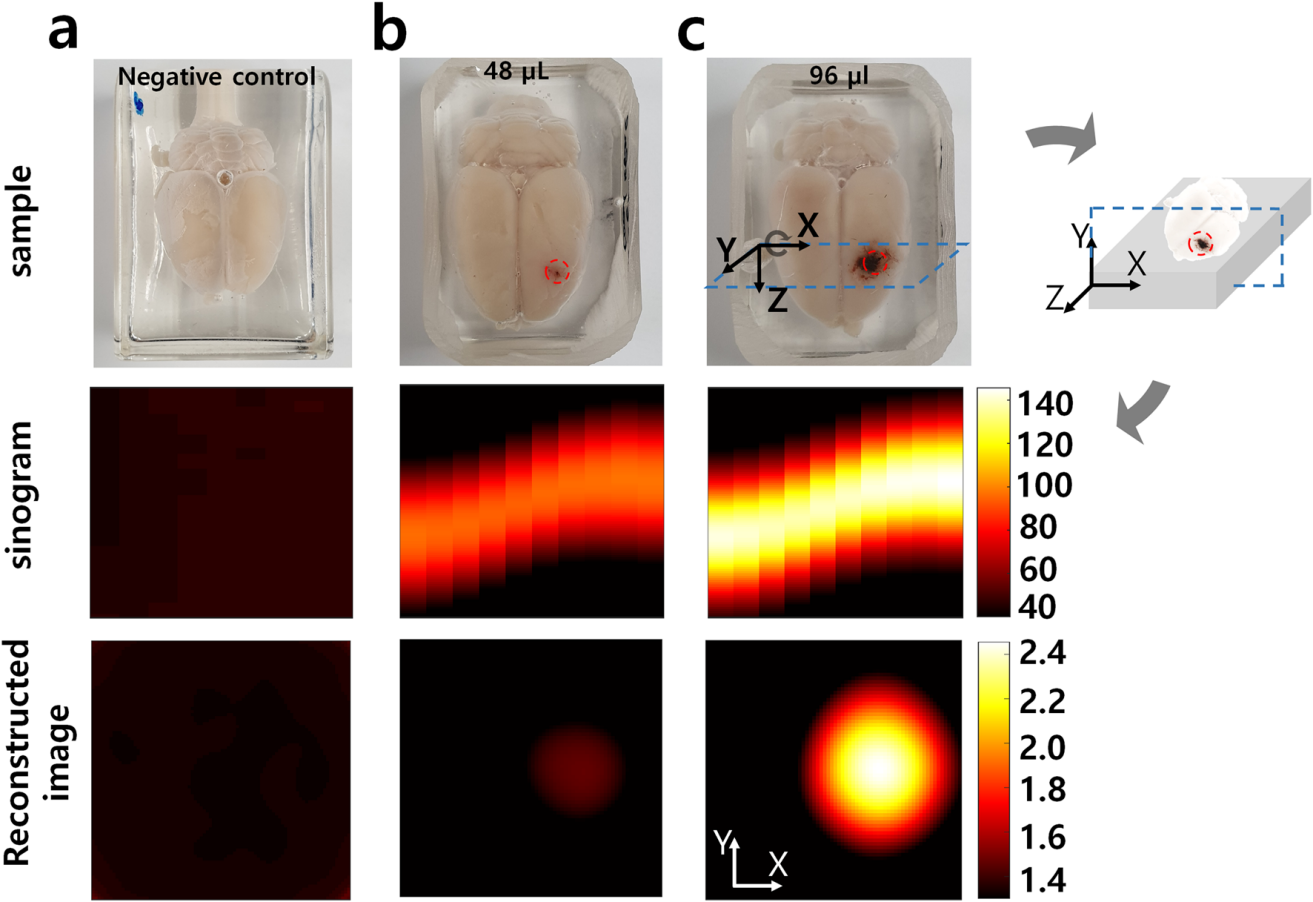
The scan of rat’s brains in which the SPIONs (dotted red circle) was injected. (**a**) negative control, (**b**) 48 $\upmu \hbox{L}$ SPIONs, (**c**) 96 $\upmu \hbox{L}$ SPIONs.

**Figure 7:**
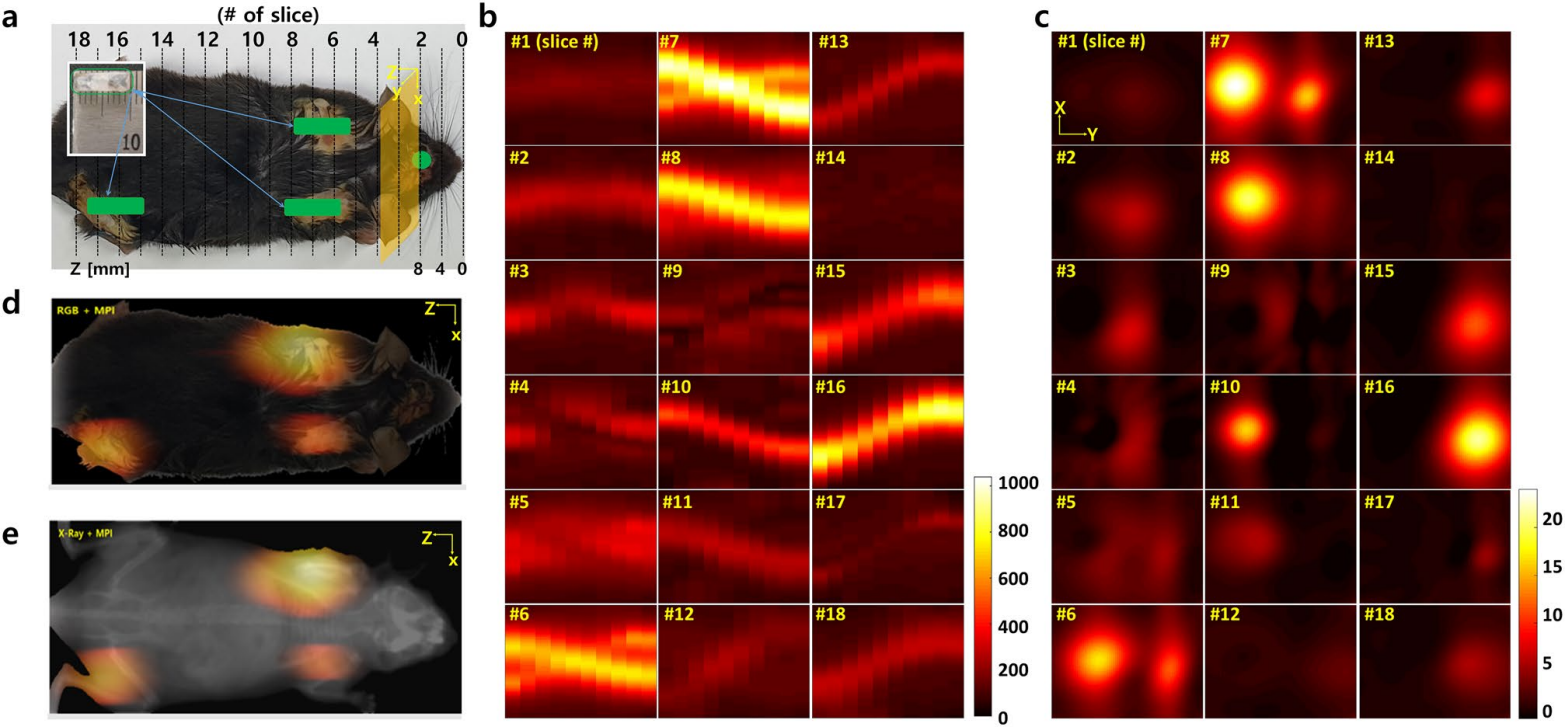
3D scan result of alive mouse. (**a**) the SPIONs-injected mouse. (**b**) sinograms. (**c**) reconstructed images. (**d**) overlay of RGB and reconstructed image. (**e**) overlay of X-ray and reconstructed image.

### Applications on biomaterials

We examined whether the SPIONs contained in the biomaterial can be imaged by the PoCT-MPI. The SPIONs used in this experiment were fluid mag-uc of Chemicell. The SPIONs (dotted red circle) were injected into the parenchyma of the rat’s brains in Fig. 6. After injection, rat’s brains were embedded in UV-resin for experiments as shown in top row of Fig. 6. A scanned image of a rat brain without SPIONs is used as a negative control image (Fig. 6a). Samples containing 48 $\upmu \hbox{L}$ and 96 $\upmu \hbox{L}$ (10 mg/ml of Fe) of SPIONs were scanned by the PoCT-MPI as shown in Fig. 6b,c. Since the sample was inserted into the +Z direction, the PoCT-MPI scanned the XY plane (dotted rectangle). Thus, the SPIONs were located in the middle of the right side on the scan plane. The reconstructed images show that the SPIONs injected in biomaterials can be imaged by the PoCT-MPI.

### Applications on mice in vivo

Finally, we performed the 3D scanning on alive mice using the PoCT-MPI. Figure 7 shows the results of the scanning on mice with the PoCT-MPI in vivo. The SPIONs used in this experiment were fluid mag-uc of Chemicell. First, a SPIONs-injected anesthetized mouse was loaded in the sample holder of the MPI scanner as shown in Fig. 7a. The 9 mm long vinyl tube shown in the upper left of Fig. 7a. are inserted into both shoulders and right leg areas through subcutaneous surgery. In addition, SPIONs were injected through the needle in the head area. For more information on the preparation of the phantom sample, refer to “Methods” section. Subsequently, a sinogram was generated for a slice perpendicular to Z direction. Since we generated the image by moving the sample in 4 mm increments in the Z direction, 18 sinograms were obtained for a mouse sample as shown in Fig. 7b. Then, the sinograms were reconstructed to 2D images by an inverse Radon transform as shown in Fig. 7c. Then, we passed the reconstruted 2D images into an *isosurface, isocat* function^20, 21^ to create a 3D volume image of sample. Finally, the slice images of the user desired directions are extracted from the 3D volume image. For visualization of the results, we superimposed the PoCT-MPI image on the photograph and X-Ray image of the sample in a top-view as shown in Fig. 7d,e. The user can then easily determine the location of the tracer or disease. Note that the method of obtaining the 3D volume image and the top view images from the reconstructed image sequence is illustrated in Fig. 10 in the “Methods” section.

### Power and temperature

The total power consumption of the PoCT-MPI is about 240 Wh. In detail, it consumes about 196 W at DC coil of FFL generator and consumes up to 44 W at the FMMD sensor. Regarding temperature, although the heat resistance temperature of the coil (heating limit) is 120 $^\circ \hbox{C}$ (Celsius is the basic unit in this paper), we kept the temperature below 70 $^\circ \hbox{C}$ in consideration of the heat resistance temperature of the bond used to wind the coil in the room temperature of 20 $^\circ \hbox{C}$ In addition, it should not exceed 36.5 $^\circ \hbox{C}$, the body temperature, within six minutes which is a scan time for a slice.

**Figure 8:**
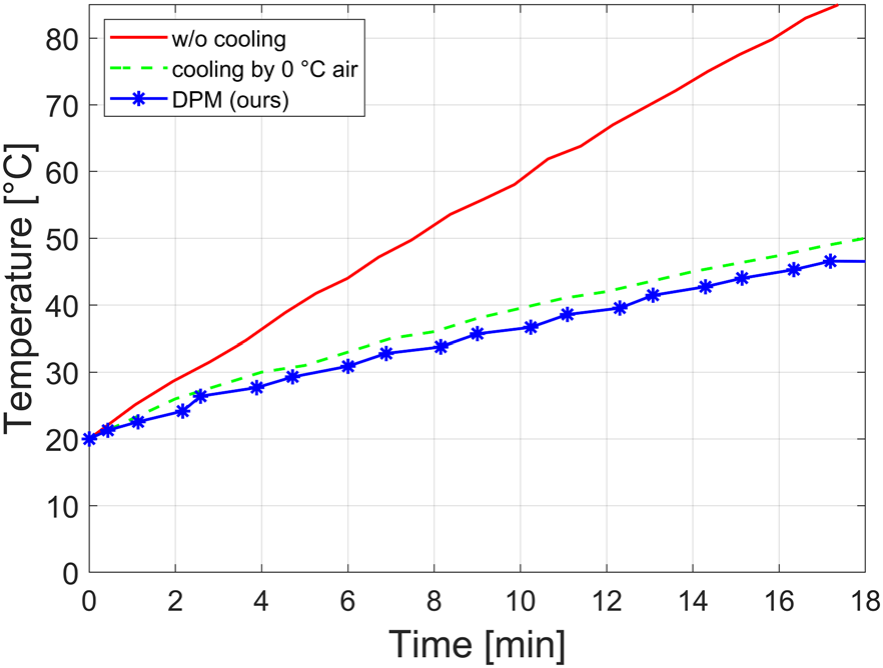
Temperature of the FFL coil.

When we were operating the PoCT-MPI at room temperature (20 $^\circ \hbox{C}$) without (w/o) cooling, the temperature of DC coil had risen 36.5 $^\circ \hbox{C}$ in 4 min, and exceeded 70$^\circ$ in 13 min after the start of equipment as shown in Fig. 8 (red line). On the other hand, we made a heat shielding box using 5 mm thin styrofoam and placed the MPI inside it. Then, we blown 0 $^\circ \hbox{C}$ (32 $^\circ$F) air coming from the air conditioner (FWC-3900, cooling capacity is 4.5 kW) into the heat shiedling box which satisfies the above temperature conditions as shown in Fig. 8 (green dotted line).

**Figure 9:**
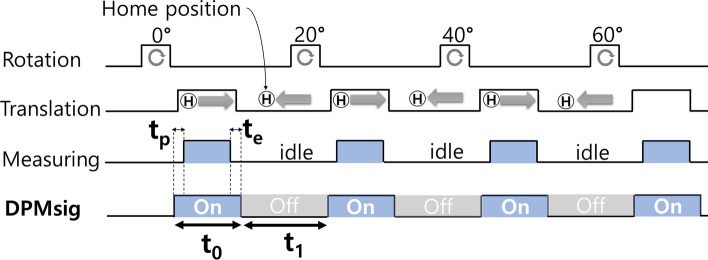
A timing diagram of DPM.

Next, the Dynamic Power Management (DPM) method was introduced in PoCT-MPI to satisfy the temperature conditions without any external cooling devices as shown in Fig. 8 (blue astarisk line). The DPM works as follows for the PoCT-MPI. Because the cables on the stage are designed to not interfere with each other, the FFL has a pause instead of rotating continuously. In particular, we observed that an idle time occurred while the FFL returned to its home position for every rotation. During this idle time, we cut off the power supplied to the FFL generator and FMMD sensor to decrease the coil temperature and reduce power consumption. In more detail, After the FFL has rotated to the current angle and translation is complete, the DPM algorithm cuts off the power supplied to the the coils. Then the coils have time to release heat while the FFL moves to the home postition of the translation and go to a next angle as shown Fig. 9. Next, power is supplied to the coil at time $t_p$ to begin scanning at the new angle. Then, after the measurement is completed, the power to coils is stopped. For the stability of the measurement, the power is cut off after $t_e$ seconds. We did several experiments to find the optimal $t_p$. Then, it was confirmed that there were no problems in imaging and heat dissipation, when $t_p$ was 500 ms and $t_e$ was 100 ms. For easy understanding, DPMsig is a kind of coil power control clock signal which stays high for $t_0$ second from a ($t_0\,+\,t_1$) period signal and its duty cycle is $(100 \times t_0) / (t_0 + t_1) \%$ as shown in the DPMsig in Fig. 9. As a result, the PoCT-MPI only consumes 120 Wh without any extra delay.

**Table 1:** Comparison with other MPI research.

Author	Method	Imaging detection limit	Power (Wh) except motor	Selection field	Moving selection field	Spartial resolution (mm)
Gleich^1^	FFP, single freq.	N.A., Resovist (d=30 nm)	N.A.	Coil	Mechanical (x,y,z)	20
Goodwill^7^	FFL, single freq.	$5\,\upmu \hbox{g}$ (Fe)*, Resovist (d=17 nm)	8,000	Magnet	Electronics(x,y), mechanical(z)	x=3.8 z=8.4
Saritas^8^	FFP, single freq.	N.A. Resovist (d=20 nm)	30,000	Magnet	Electronics(x,y), mechanical(z)	x=2 y=2
Zheng^9, 11^	FFP, single freq.	5.4 ng (Fe), Resovist (d=20 nm)	N.A.	Magnet	Electronics(x,y), mechanical(z)	x=5 y=5
Yu^12, 13^	FFL, single freq.	**3 ng** (Fe), Resovist (d=20 nm)	12,300 (peak)	Magnet	Electronics(x,y), mechanical(z)	**x=1** **y=1** **z=0.7**
M.Graeser^10^	FFP, single freq.	$2\,\upmu \hbox{g}$ (Fe), Perimag, $d=130$ nm	240	Iron core magnet	Electronics(x,y), mechanical(z)	x=6 y=6 z=28
**Ours**	FFL, FMMD	1.3 $\upmu \hbox{g}$ (Fe), Synomag, $d=50$ nm	**120**	Hybrid (coil, magnet)	Mechanical (x,y,z)	x=5.4 y=5.4 z=15

Bold letters represent the best results.

*Note that the lowest sensitivity was not claimed in [7], but we were able to estimate the lowest sensitivity from the darkest region near a tail in proportion to the fiducial brightness they used in Fig. 11 of [7].

## Discussion

We proposed a compact, low-power 3D MPI device using a hybrid type selection field and FMMD sensor. In this section, we will compare and analyze the advantages and disadvantages of previous MPI research and ours through Table 1. Yu *et al.*^12,13^ imaged only 3 ng (Fe) of Resovist which is the lowest detection limit so far. And its spatial resoution is $1 \times 1 \times 0.7$ mm for x, y, z directions. M.Graeser^10^ developed very low power 3D MPI which consume 240 Wh. On the other hand, we succeeded in imaging $1.3\,\upmu \hbox{g}$ (Fe) of SPIONs with hydrodynamic diameters of 50 nm using PoCT-MPI. And our spatial resoution is $5.4 \times 5.4 \times 15$ mm for x, y, z directions. The resolution of the x and y directions can be improved without additional power consumption by increasing the gradient field using additional permanent magnets of the PoCT-MPI. Regarding the resolution in the Z direction, adding only the magnet in the Z direction without increasing the power of the DC coil increases the asymmetry of the shape of the FFL. Therefore, increasing the strength of the magnet can improve the resolution in the Z direction, but it causes a negative effect of crushing the shape of the FFL from circular to elliptical. At this time, in order to keep the shape of the FFL circular, the number of turns of the coil and the amount of current may be increased. This relationship is a trade-off between power consumption and spatial resolution. Therefore, it is a matter of choice depending on the application.

In sinograms, discontinuities are observed in the horizontal direction due to the large angular resolution, 20$^\circ$. Rotating sparsely has the advantages of a speeding up and a less exposure of body to medical equipment. However, the sparse sinogram is likely to cause artificial effects in the reconstructed image. To alleviate these artifacts, traditional image processing or neural networks can be used^22^, which are beyond the scope of this paper.

For the “Applications on mice in vivo” experiment, the intensity around the right shoulder is lower than that of the left shoulder in images of slide from 6 to 8 in Fig. 7c. On the other hand, the intensity around the right leg has a similar value to that of the left shoulder image in the slide 16. Since tubes containing the same amount of SPIONs are inserted into the both shoulders and the right leg, we can notice that there is no serious sensitivity difference between left and right side in the equipment. However, since the fluid mag-uc in which cores are not coated is used for the mice experiment, there is a possibility that the aggregated cores have lost super-paramagnetic properties while staying at body temperature for three days. And as can be seen from slide 1 to 3, SPIONs injected around the head are weakly measured, while strong signals is observed around the left side of abdominal region as shown in slide 10. It is possible that the SPIONs injected into head without tube were absorbed by the liver. However, the medical analysis of this phenomenon is not covered in this paper.

## Conclusion

We introduced a low-power compact MPI that can be used for PoCT. In order for MPI technology to be widely used for PoCT, it is necessary to preserve high SNR while reducing power consumption with small size of system. To secure the PoCT-MPI, we applied FMMD and DPM method to MPI, and proposed hybrid FFL generator. The PoCT-MPI can scan a $60 \times 40 \times 40\,\hbox{mm}^3$ phantom sample while consuming less than 120 Wh of power. It can image $0.13 \upmu \hbox{L}$, 10 mg/mL (Fe) of SPIONs with 50 nm in hydrodynamic diameters. We also succeeded in 3D imaging of SPIONs injected in rodents in vivo with the PoCT-MPI.

We are working on increasing the FMMD sensor’s diameter to 50 mm to scan larger samples. We are also planning a study on battery-powered MPI systems that further reduce power consumption. Finally, we are preparing studies to image the location of cancer in rodents using the PoCT-MPI.

## Methods

### SPIONs

We used two types of SPIONs to determine the effect of particle’s coating on the measurement. In short, the PoCT-MPI was able to detect both coated and uncoated particles. First, we used 50 nm dextran coated SPIONs in sensitivity resolution experiments. Synomag-d, product no. 104-00-501 from Micromod, exhibits excellent properties as tracer for MPI system and is suitable for hyperthermia applications^23^. Its iron concentration is $10\,\upmu \hbox{g}/\upmu \hbox{L}$ of Fe and the solid concentration is $25\,\upmu \hbox{g}/\upmu \hbox{L}$. Next, we used uncoated Chemicell’s uc/a type (fluid mag-uc/a, Chemicell GmbH, Berlin, Germany) for the experiments of biomaterials and in vivo. Its iron concentration is $10\,\upmu \hbox{g}/\upmu \hbox{L}$ of Fe and the weight of volume is $25\,\upmu \hbox{g}/\upmu \hbox{L}$. The hydrodynamic diameters of SPIONs are all 50 nm size products for easy drainage^24^.

### Sinograms

Considering the structure of the FFL generator and shifter, the FFL of the PoCT-MPI rotates clockwise from 0$^\circ$   180$^\circ$ in 20$^\circ$ increments around the Z axis, while translating (moving) $\pm 40\,\hbox{mm}$ in the X direction for each rotation for a sinogram. Then, FFL repeats this process in 10 mm increments in the Z direction, producing 18 sinograms as shown in Fig. 7b.

However, the Radon algorithm^18^ assumes that the ray rotates counterclockwise and translates from left to right. Thus, the angle $\theta$ needed to be corrected to 180$^\circ$− $\theta$ and the translation index needed to be changed from $\pm 40$ to ${\mp } 40$ in order to obtain the same sinogram as the algorithm as shown in Fig. 3.

### Image reconstrution

We reconstructed images from the Radon transform of which result is sinogram, using a single iteration of the Simultaneous Algebraic Reconstruction Technique (SART) algorithm^18^. And this algorithm has been implemented well with a python language^19^.

**Figure 10:**
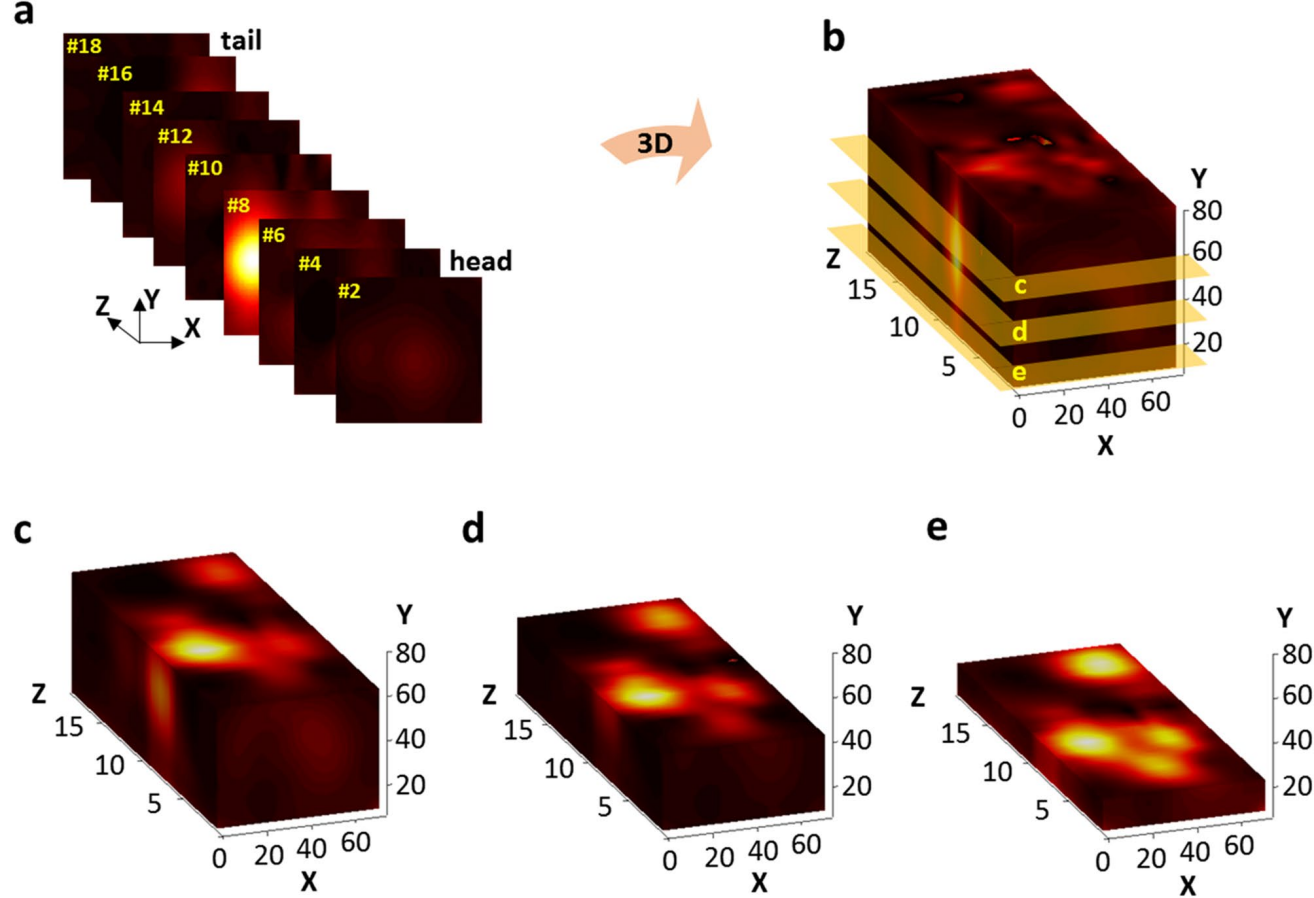
2D and 3D volume images of sample. (**a**) image sequence of XY plane, (**b**) 3D volume image, (**c**)–(**e**) 2D images (in top) when Y is 60 mm, 40 mm and 20 mm.

Figure 10 shows that the XY scan results of the PoCT-MPI can be merged to a 3D volume and then a 2D image can be generated by cutting in a desired direction.

Figure 10a are 2D image sequence of the XY plane, and they were packed in the Z direction to create a 3D volume image (**b**). The 3D volume image has a matrix form by interpolating the values of all internal points. Therefore, if one of the coordinate values of X, Y, and Z is selected, the 2D image can be restored using *isosurface, isocap* functions of Matlab^20,21^. For example, if you set the Y coordinate of Fig. 10b to 60 mm, 40 mm and 20 mm, then you can create top view images of the XZ plane such as (**c**), (**d**) and (**e**), respectively.

### Preparing rat’s brains

In vivo animal experiments were performed in accordance with the ethical guidelines of the National Institutes of Health Guide for the Care and Use of Laboratory Animals (NIS Publication No. 80-23, revised 1996) under approval of the Eulji University Institutional Animal Care and Use Committee (No. EUIACUC17-10). For phantom sample preparation, adult male Sprague-Dawley rats (n = 5, body weight, $280 \sim 320\,\hbox{g}$; Charles River Lab. Wilmington, DE, USA) were anesthetized intraperitoneally with ketamine (70 mg/kg body weight) and xylazine (8 mg/kg body weight) and perfused through ascending aorta with 300 mL of 4% paraformaldehyde in 0.1 M phosphate buffered saline. Brains were removed and transected horizontally into the upper and lower parts.

Small vinyl tubes (3.0 mm in diameter and 10.0 mm in height) containing $48\,\upmu \hbox{L}$ and $96\,\upmu \hbox{L}$, ($10\,\upmu \hbox{g}/\upmu \hbox{L}$ of Fe) of SPIONs were inserted into the upper part of brain parenchyma and the upper and lower parts of the brain were joined. The SPIONs used in this study was Chemicell’s UC/A type (Fluid MAG-UC/A, Chemicell GmbH, Berlin, Germany). Brains was placed in a plastic box (20.0 mm wide, 35.0 mm long and 20.0 mm high) and 7.0 mL of ultraviolet curing resin (UV resin Hard Type, Padico, Tokyo, Japan) was applied and polymerized by irradiating 265 nm ultraviolet ray for 10 minutes. The brain inserted with vinyl tube containing deionised water was used as the negative control.

### Preparing alive mice

Male C57BL/6 mice (n = 3, body weight, 2–25 g; Jackson Laboratories, Bar Harbor, ME, USA) were used for in vivo 3D MPI Sensor experiment. Under deep anesthetization with ketamine (14 mg/kg body weight) and xylazine (1.6 mg/kg body weight), mice were secured in a stereotaxic apparatus (Stoelting Co., Wood Dale, IL, USA). The skull was exposed by a midline incision and a small hole was made with a dental drill in the right side of the skull 0.8 mm posterior to the bregma and 1.6 mm lateral to the midline. A 26-gauge needle attached to $10\,\upmu \hbox{L}$ Hamilton syringe (Hamilton Co., Reno, NV, USA) was lowered through the hole to a depth of 2.0 mm from the skull. Six $\upmu \hbox{L}$ of SPIONs was administered with micro-infusion pump (KD Scientific Inc., Holliston, MA, USA) at a rate of $0.5\,\upmu$L/min.

The needle was left in place for a further 5 min before slowly retracting. The injection procedure was repeated more two times in the adjacent region (0.5 mm left and right from the original injection site), which made the total volume of 18 $\upmu \hbox{L}$ ($10\,\upmu \hbox{g}/\upmu \hbox{L}$ of Fe)) of SPIONs was administered into the brain. Under consecutive anesthetization, small skin incisions on the bilateral scapular region and unilateral (right) pelvic region were made and vinyl tubes containing $18\,\upmu \hbox{L}$ ($10\,\upmu \hbox{g}/\upmu \hbox{L}$ of Fe) of SPIONs were inserted subcutaneously. The quantities of tracer have chosen based on the sensitivity and resolution of our MPI system obtained from phantom sample experiments. Mice were scanned three days after surgery for a stable experiment.

## System implementation

### Frequency mixing magnetic detection sensor

**Figure 11:**
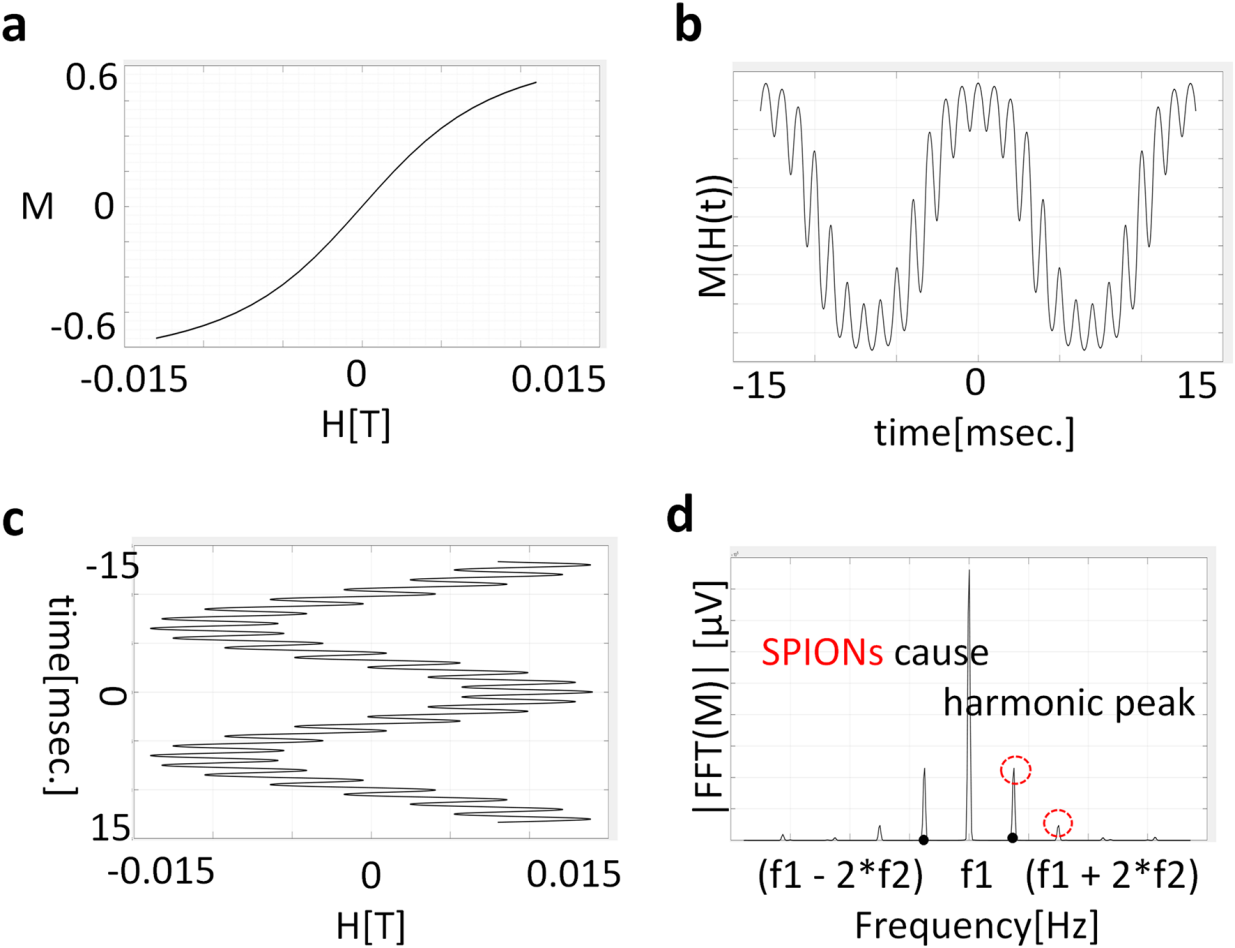
Simulation of the FMMD method. (**a**) The nonlinear magnetization curve of SPIONs. (**b**) The magnetic fields consisting of two frequency components $f_1$ and $f_2$. (**c**) A distorted response signal. (**d**) A spectrum analysis of response signals with fast fourier transformation (FFT).

The FMMD method applies an AC magnetic field with two different frequencies to the SPIONs sample to improve the SNR of the received signal unlike other MPI studies^1,7,8,10,25^. SPIONs with nonlinear magnetization as shown in Fig. 11a are exposed to magnetic fields consisting of two frequency components $f_1$ and $f_2$ as shown in Fig. 11c. Then, due to the non-linear magnetization characteristics, the response signal would be distorted as shown in Fig. 11b. The particles saturates at higher fields, leading to higher harmonics and frequency mixing components in the spectrum analysis such as fast fourier transformation (FFT) of response signal,**c**, as shown in Fig. 11d. The magnetization M of superparamagnetic magnetic particles may be approximated by the Eq. (1)

**Figure 12:**
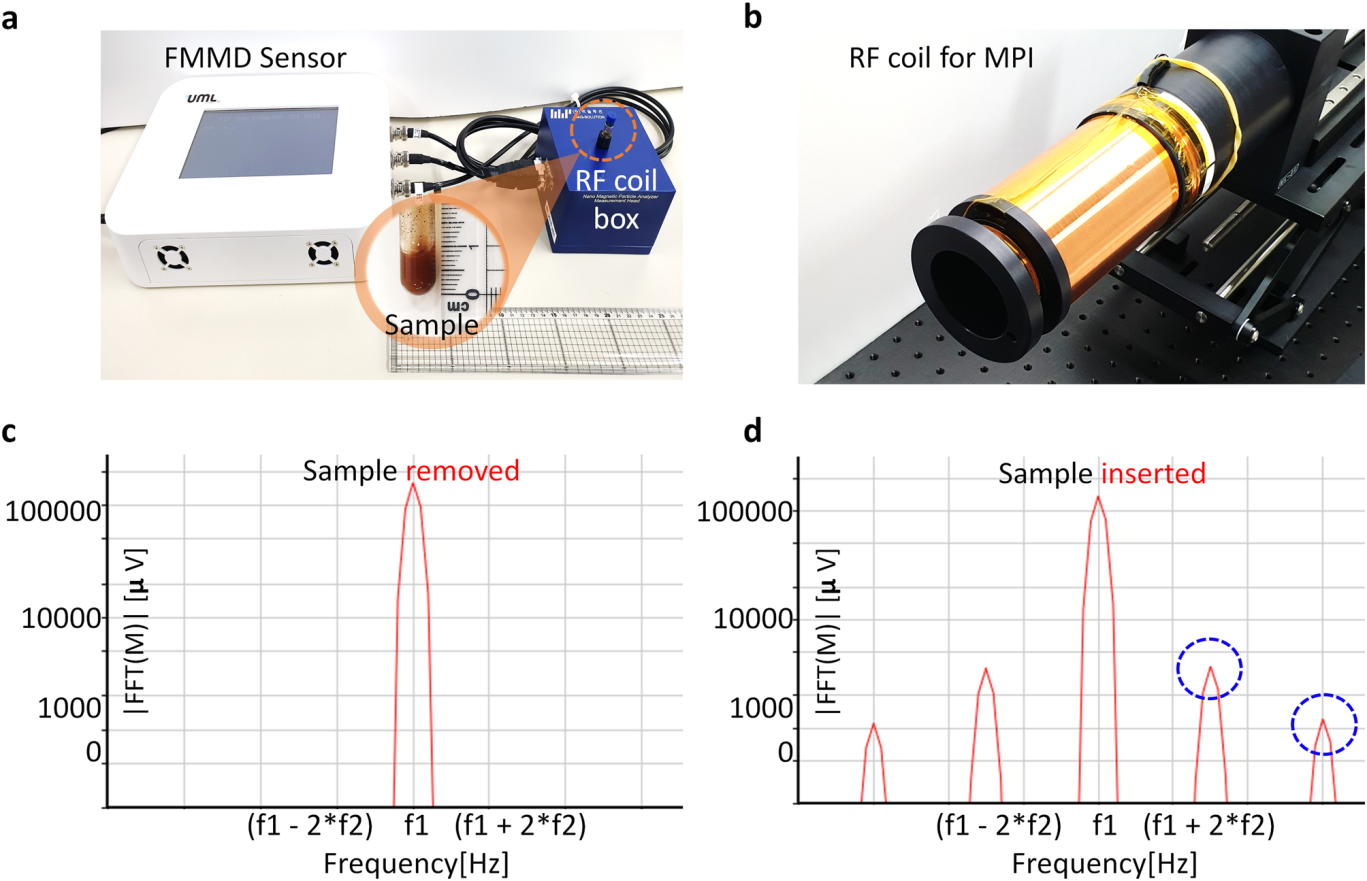
FMMD sensor. A radio frequency (RF) coil box in (**a**) was replaced with (**b**) for PoCT-MPI. (**a**) The FMMD sensor. (**b**) 40 mm (inner diameter) RF coil for PoCT-MPI. (**c**) The output with no SPIONs. (**d**) The output with SPIONs.

1 $$\begin{aligned} M(\upmu H) = M_s \cdot {\mathcal {L}}\left( \dfrac{m_0 \upmu _0 H}{k_B T}\right) . \end{aligned}$$

where ${\mathcal {L}}(x)$ is the so-called Langevin function^26^:

2 $$\begin{aligned} {\mathcal {L}}(x) = coth(x) - \left( \dfrac{1}{x}\right) . \end{aligned}$$

$$m_0$$ is the magnetic moment in Am$$^2$$ of a single magnetic particle, $$\upmu _0 = 4\pi \times 10^{-7}$$ Vs/Am denotes the vacuum permeability, $$k_B$$ is the Boltzmann constant, *T* is the temperature in Kelvin, $$M_s$$ is the saturation magnetization of the particles, and $$x=m_0\upmu _0H/k_BT$$ denotes the dimensionless (scaled) magnetic field. In case the magnetic particles are exposed to a magnetic field consisting of two distinct excitation frequencies $$f_1$$ and $$f_2$$(with $$f_1 > f_2)$$:

3 $$\begin{aligned} \upmu _0 H(t) = B_0[A_0 + A_1 sin (2 \pi f_1 t) + A_2 sin(2 \pi f_2 t)], \end{aligned}$$

where, $B_0$ is the scale factor of the magnetic field, $A_0$ is the DC magnetic field, and $A_1$ and $A_2$ are the amplitudes of high frequency $f_1$ and low frequency $f_2$, respectively.

The third order mixing component $f_1 + 2f_2$ of Langevin function’s Taylor expansion at a field *x* near 0 may be written as

4 $$\begin{aligned} M_3(t) = M_s \frac{A_1A^2_2}{8} \cdot {\mathcal {L}}^{(3)}(x) \times cos[2\pi (f_1 + 2f_2)t], \end{aligned}$$

The approximated Eq. (4) is valid only in the limit of small excitation amplitudes. The physical meaning of Eq. (4) is the probe value indicating the strength of the SPIONs concentration. When using one frequency as in the traditional methods, the parameters $f_1$ and $f_2$ become identical, i.e. $f1 = f2$. Since $M_3 (t)$ is scaled at a ratio of $A_1 A^2_2$ for the frequency $(f_1 + 2f_2)$ component in the SPIONs concentration, the higher $A_1 A^2_2$, the higher the SNR performance.

**Figure 13:**
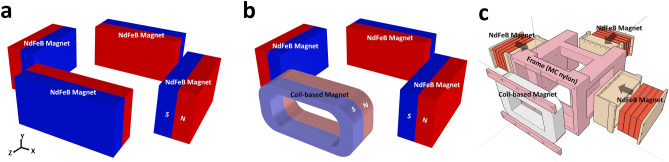
The proposed FFL generator of the PoCT-MPI. (**a**) A quadrupole magnet^27^ based FFL generator, (**b**) The proposed hybrid FFL generator in which magnets in one side of (**a**) are replaced with a coil-based electromagnet so that the sample can be inserted inside in the Z-axis. (**c**) A computer-aided design (CAD) of PoCT-MPI’s FFL generator. The figure was created using Rhino 6.0. (https://www.rhino3d.com/).

In summary, the scale factor of a single frequency is $A_1^3$, the scale factor of the FMMD method is $A_1 A ^ 2_2$. To increase $A_1$, one can need high AC current amplifiers and coils made from stranded litz wire which is able to minimize skin effects^28^.

On the other hand, for $A_2$ at low frequencies, an impedance caused by a coil inductance is low, so that high SNR can be realized with normal AC amplifiers and single wire coils.

In terms of low power and portability for PoCT-MPI, it is important to transmit and receive signals in a single control device, because many common semiconductor chips for a device, such as regulators, processors and memories can be shared.

We proposed FMMD sensor device capable of transmitting, amplifying, receiving and processing two-channel sinusoidal analog signals in one device as shown in Fig. 12a. The measurement head (blue cube box) has a small coil with 8 mm diameter to measure the magnetization properties of small sample with SPIONs as shown in Fig. 12a. A new coil with 40 mm diameter was designed to measure samples of rodent size as shown in Fig. 12b. In addition, if necessary, it can be linked with an external power amplifier to generate a peak-to-peak 20 mT magnetic field at the center of our designed FFL generator. To measure a sample with a diameter of 40 mm, we use AE Techron’s AE7224 power amplifier, which amplifies the sinusodal signal at $\pm 60$ V at 1 A. The millivolt-level raw analog signal obtained from the receiving coil is input to the sensor and then it is filtered and amplified up to $\pm 4$ V.

**Figure 14:**
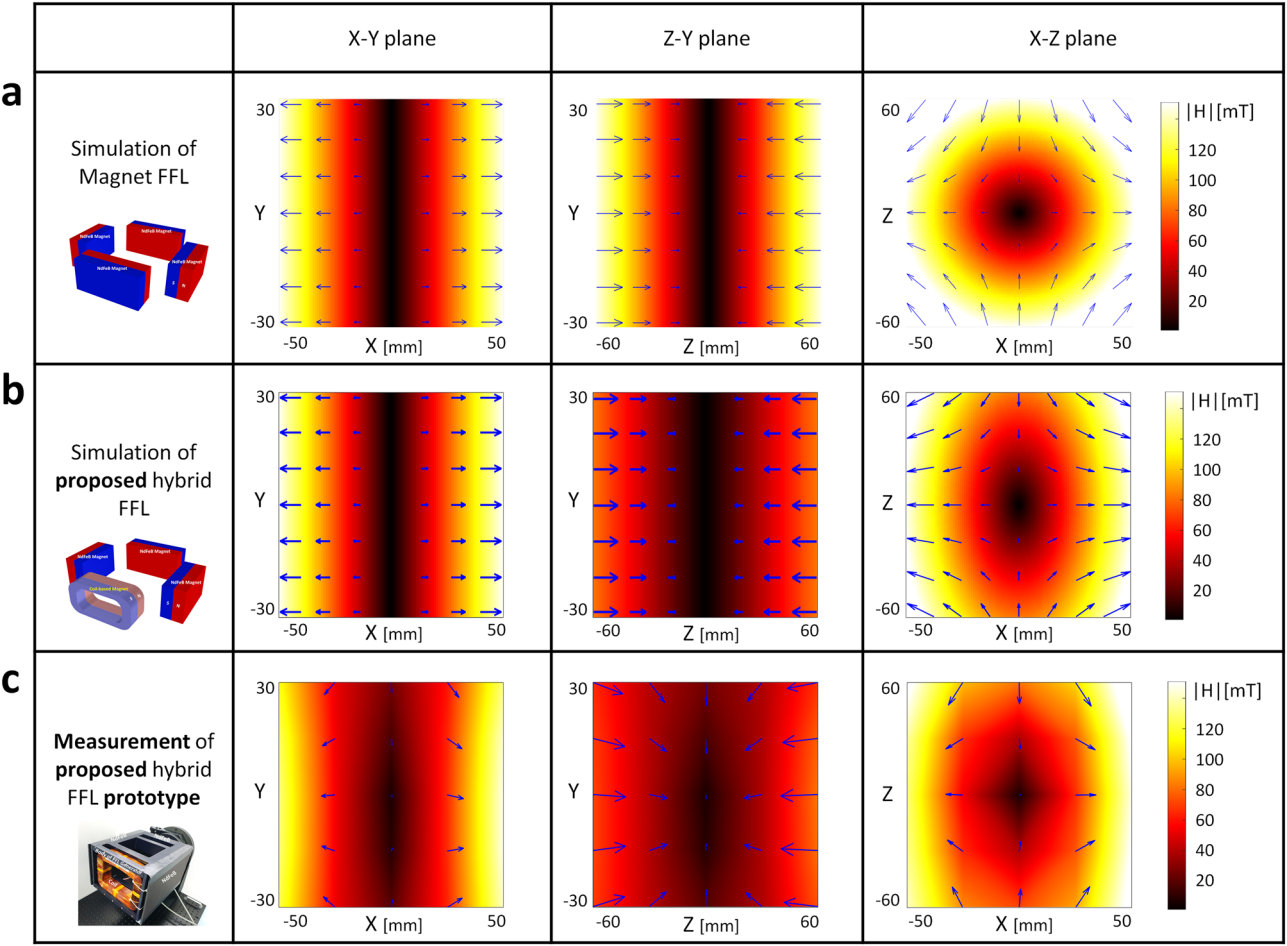
Comparing Selection Field between quadrupole and hybrid magnets. (**a**) are magnetic fields of quadrupole with a gradient of $(G_x,G_y,G_z) = (2.9, 0, -2.9)$ T/m. (**b**) are the magnetic fields of the proposed hybrid magnet in which amples can be fed into it, with gradient $(2.9, 0, -1)$ T/m. (**c**) shows the measuring result of the manetic field of the prototype of the hybrid magnet.

Figure 12c is the result of frequency analysis of the output signal in zeroing state without a sample. The high and the low frequency are applied at $7,000\,\hbox{Hz}\,(f_1)$ and $75\,\hbox{Hz}\,(f_2)$ and for the amplitude in Tesla, $A_1$ is about 2 mT for 7,000 Hz and $A_2$ is about 15 mT for 75 Hz. Note that the frequency values used here are empirical hyperparameters selected by consideration of coil impedance matching and repeated experiment. When a sample containing SPIONs is not inserted, then no harmonic peaks occurs except fundamental frequencies as shown in Fig. 12c.

On the other hand, when the sample containing SPIONs is loaded into the measurement coil, we can observe harmonic peaks in the software of FMMD sensor. The voltage peaks of the harmonic frequencies such as 7,150 $\hbox{Hz} (= f_1 + 2 f_2)$ are proportional to the concentration of the SPIONs as shown in Fig. 12d. This value is also proportional to $M_{s} \frac{A_{1} A_{2}^{2}}{8} \cdot {\mathcal {L}}^{(3)}(x)$, the magnitude term of Equation 4. If the frequency and amplitude of the input signal are constant, it is a voltage proportional to $M_{s} \cdot {\mathcal {L}}^{(3)}(x)$, so it can be used as a measurement signal for the distribution of SPIONs samples.

### Hybrid FFL generator

Signals obtained from the FMMD sensor are the integral of harmonic signals from all the SPIONs existed in the sample. Therefore, the combined signals need to be separated into spatial signals by introducing a selection field in the magnetic field excitation process^1,7,25^. Since FFL can achieve a speedup by the number of pixels corresponding to the line length over FFP, we used FFL as the selection field^8^.

Traditional FFL generators can be implemented with quadrupole magnets^27^ as shown in Fig. 13a. In the quadrupole magnet, two magnets face each other with the same polarity, **S**, in X direction. The other pair of magnets faces each other with **N** polarity in Z direction. Then, this combination of magnets produces a long FFL in the Y direction at the center of XZ plane, as shown in Fig. 14a. However, this structure has the disadvantage that the sample is situated in a closed space. Note that it is difficult to scan in the Z direction when the sample is inserted in the Y direction, since the direction FFL and the insertion of samples are parallel to each other. To make the sample inlet in the middle of the magnet, a torus-shaped magnet could be consider, but it has a problem that the magnetic field is not homogeneous in the gradient field region. In detail the magnetic field of the inner surface exists regardless of whether it is square or circular, so that the direction of the magnetic field is difficult to be uniform at the sample position in a donut-shaped magnet. After all, a magnet in the form of a solenoid coil is the solution.

**Figure 15:**
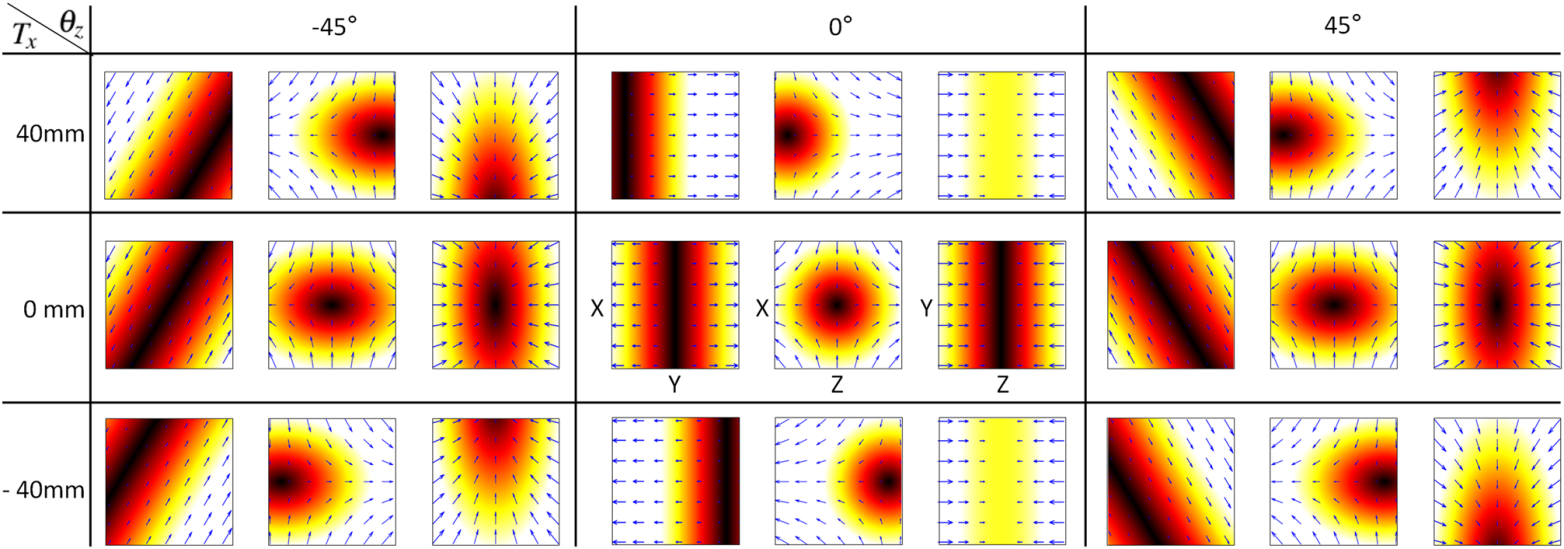
Rotation and translation of selection field. Each elements consist of three slice of plane,which are XY, XZ, Z-Y plane, respectively. Nine combinations consisting of Z-axis rotation ($-45^\circ$, 0$^\circ$, 45$^\circ$) and translation $(-40,0,40)$ mm in the rotated X-axis direction is also displayed.

Figure 13b shows the hybrid generator proposed to solve this problem in which the permanent magnet at one side of the Z-axis is replaced with a coil-based electromagnet, so that the sample can be fed along the Z direction. Figure 13c is the computer-aided design of **b**.

Note that due to the difference in characteristics between the magnet and the coil, XZ plane may be asymmetric in the Z direction when $|G_x| \ne |G_z|$ as shown in Fig. 14b,c, where $G_{x}:=\frac{\partial H_{x}}{\partial x},G_{z}:=\frac{\partial H_{z}}{\partial z}$ are the gradients of each direction. Figure 14a shows the simulation results of the magnetic field of a quadrupole magnet based FFL generator. To imagine the shape of the FFL, you can think of a pencil with a long circular cross section that stands in the Y direction. On the other hand, Fig. 14b shows a long elliptic cross section on the XZ plane because the gradient field in the Z direction is smaller than that in the X direction. The value of $G_x$ and $G_z$ is 2.9 and $-2.9$ for quadrupole magnet and 2.9 and $-1.0$ for hybrid structure, respectively, where $G_x$ and $G_z$ are from Equation 6. Figure 14b,c show almost identical results of the simulated magnetic fields (**b**) and that of measured magnetic fields (**c**). To measure the internal magnetic field of the FFL generator, 150 (or $5 \times 5 \times 6$) positions are set, then the magnetic intensity are measured at each position (intermediate values are interpolated with bilinear method).

The frame of the FFL generator is made of monomer casting (MC) nylon material. measuring $20 \times 33 \times 45\,\hbox{cm}^3$ and weighing less than 40 kg. The maximum size of sample (cylindrical shape) can be loaded is 40 mm in diameter and 60 mm in length.

### Shifting FFL for sinogram

We need to mathematically model the selection field before moving it. In modeling the rotation and translation of the FFL generator, we introduce homogeneous coordinates^29,30^ in which the last element increases by one dimension for ease of computation. Assuming that the gradient field of the selection field is linear, the gradient matrix ${{\varvec{H}}}_0^{{\rm S}}({{\varvec{r}}})$ represents the magnetic field of the selection field in Fig. 13a. Accordingly, Equation (5) in Euclidean space can be transformed into homogeneous coordinates.

5 $$\begin{aligned} {{\varvec{H}}}_0^{{\rm S}}({{\varvec{r}}}_0)&= \left( \begin{array}{ccc}{G_{x}}&{} {0} &{} {0} \\ {0} &{} {G_{y}} &{} {0} \\ {0}&{} {0} &{} {G_{z}}\end{array}\right){{\varvec{r}}}_0 \\&= \left( \begin{array}{ccc}{G_x}&{} {0} &{} {0} \\ {0} &{} {0} &{} {0} \\ {0}&{} {0} &{} {-G_x}\end{array}\right) {{\varvec{r}}}_0\\&= \left( \begin{array}{ccc}{2.5} &{} {0} &{} {0} \\ {0} &{} {0} &{} {0} \\ {0} &{} {0} &{} {-2.5}\end{array}\right) {{\varvec{r}}}_0 = G_0 r_0, \end{aligned}$$

where ${{\varvec{r}}}_0 = [x,y,z]^{T}$ is the position vector and $G_{x}:=\frac{\partial H_{x}}{\partial x},G_{y}:=\frac{\partial H_{y}}{\partial y}$, and $G_{z}:=\frac{\partial H_{z}}{\partial z}$ are the gradients of each direction.

**Figure 16:**
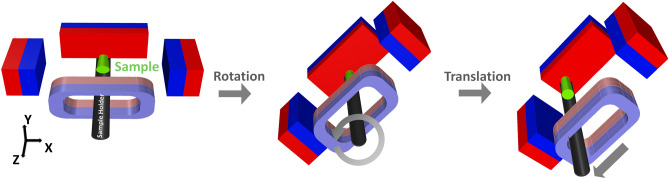
Shifting FFL generator. The FFL generator shifts with the sample fixed. The figure was created using Rhino 6.0.

Since the new *G* matrix represents the selection field in the FFL generator, one can multiply position vector $\underline{\mathbf{r }}$ by *G* to get the H field vector $H ^ S (r)$ of the point. For the simulation prior to prototyping, we define the rotation matrix $R_z$ to rotate the FFL generator by $\theta$ around the Z axis as Eq. (7).

Then, a translation matrix T is defined to shift $t_x$, $t_y$, and $t_z$ in X, Y, and Z directions. Next, the H field matrix ${{\varvec{H}}} ^ {{\rm S}} _ {R,T} (\varvec{{\bar{r}}})$ is calculated by Eq. (9).

We can see that the rotation of a selection field consisting of gradient fields by Eq. (9) has the same effect as the FFL scan as shown in Fig. 15. It shows the result of FFL scanning simulation over $(0 \le \theta _z \le 180)^\circ$, $(-40\,\hbox{mm} \le t_x \le 40\,\hbox{mm})$ sections. Note that an FFL scan is performed on the 2D slice of the sample in the XY plane perpendicular to the Z-direction.

6 $$\begin{aligned} {{\varvec{H}}}^{ {\rm S}}({{\varvec{r}}})&= \left( \begin{array}{cccc}{G_{x}} &{} {0} &{} {0} &{} {G_{x_{os}}} \\ {0} &{} {G_{y}} &{} {0} &{} {G_{y_{os}}}\\ {0} &{} {0} &{} {G_{z}} &{} {G_{z_{os}}} \\ {0} &{} {0} &{} {0} &{} {1}\\ \end{array}\right) {{\varvec{r}}}\\&= \left( \begin{array}{cccc}{2.5} &{} {0} &{} {0} &{} {0} \\ {0} &{} {0} &{} {0} &{} {0}\\ {0} &{} {0} &{} {-1} &{} {0} \\ {0} &{} {0} &{} {0} &{} {1}\\ \end{array}\right) {{\varvec{r}}} = G r, \end{aligned}$$

where $\underline{\mathbf{r }}$, $[x,y,z,1]^{T}$, is a position vector in 3D space, and $G_{x_{os}}$, $G_{y_{os}}$, and $G_ {z_{os}}$ are constant offset fields in the corresponding directions, respectively. The offset fields are introduced to indicate how far the center of the each gradient field is from the origin of the FFL generator.

7 $$\begin{aligned} \varvec{R_z}({\theta _{z}})= & {} \left( \begin{array}{cccc}{\cos \theta _z} &{} {-\sin \theta _z} &{} {0} &{} {0} \\ \sin \theta _z &{} \cos \theta _z &{} {0} &{} {0}\\ {0} &{} {0} &{} 1 &{} {0} \\ {0} &{} {0} &{} {0} &{} {1}\\ \end{array}\right) . \end{aligned}$$

8 $$\begin{aligned} {{\varvec{T}}}({{t_x,t_y,t_z}})= & {} \left( \begin{array}{cccc}{1} &{} {0} &{} {0} &{} {t_x} \\ {0} &{} {1} &{} {0} &{}{t_y}\\ {0} &{} {0} &{} 1 &{} {t_z} \\ {0} &{} {0} &{} {0} &{} {1}\\ \end{array}\right) . \end{aligned}$$

9 $$\begin{aligned} G_{rot}= & {} R \times G\nonumber \\ T_{rot}= & {} R \times T\nonumber \\ G_{R,T}= & {} G_{rot} \times T_{rot}^{T}\nonumber \\ {{\varvec{H}}}^{ {\rm S}}_{R,T}(\varvec{{\bar{r}}})= & {} G_{R,T} \times {\bar{r}} , \end{aligned}$$

where ${\bar{r}}$ is $[x,y,z,1]^T$ and $\times$ is a matrix multiplication. Figure 16 shows that the three-dimensional selection field can be rotated and translated by Eq. (9). For the sake of understanding, nine combinations consisting of Z-axis rotation ($-45^\circ$, 0$^\circ$, 45$^\circ$) and translation $(-40,0,40)$ mm in the rotated X-axis direction are also displayed. The combination in the middle shows the default selection field as $(t_x, \theta _z) = (0,0)$. As the value of translation $t_x$ changes, the FFL of the XZ plane and the XY plane can be observed to move linearly. Also, as $\theta _z$ changes, we can see that the FFL standing in the XY plane rotates to an angle.
